# Bone Tissue Engineering: Scaffold Design Principles, Biomaterial Advances, and Strategies for Functional Regeneration and Clinical Translation

**DOI:** 10.3390/bioengineering13050514

**Published:** 2026-04-29

**Authors:** Naznin Sultana

**Affiliations:** Texas Undergraduate Medical Academy, School of Public and Allied Health, Prairie View A&M University, Texas A&M University System, Prairie View, TX 77446, USA; nasultana@pvamu.edu

**Keywords:** bone tissue engineering, scaffold design, osteoconduction, regenerative medicine, bone graft substitutes

## Abstract

Bone is a hierarchically organized composite material with unique mechanical properties and an intrinsic regenerative capacity that conventional repair strategies, including autografts, allografts, xenografts, and metallic or ceramic implants, fail to fully replicate due to donor scarcity, immunogenicity, mechanical mismatch, and poor long-term integration. Bone tissue engineering (TE) offers a biologically informed alternative by integrating osteoconductive scaffolds, osteogenic progenitor cells, and osteoinductive signaling molecules into a unified regenerative framework. Unlike existing reviews that evaluate these components in isolation, this review provides a mechanistically integrated analysis that repositions scaffold design as a biologically instructive platform whose topography, stiffness, porosity, and surface chemistry collectively govern cell adhesion, mechanotransduction, osteogenic differentiation, and extracellular matrix remodeling. Critically, it moves beyond cataloging materials and fabrication approaches to evaluate how specific scaffold features drive biological outcomes and to identify frequently understated limitations, including polymer-ceramic degradation kinetics and the inadequacy of small-animal models for clinical translation. By synthesizing advances in biomaterials, additive manufacturing, and smart scaffold technologies within this integrative framework, this review provides researchers and clinicians with a structured framework for evaluating emerging strategies and prioritizing future directions in functional bone regeneration.

## 1. Introduction

Bone is a highly organized composite material with unique mechanical properties, which can be replicated using synthetic composites [1]. As a fundamental component of the human skeletal system, bone provides structural support and plays a critical role in maintaining body integrity. To address bone defects, numerous replacement materials and techniques have been developed. One of the most remarkable features of bone is its intrinsic ability to regenerate and remodel without limitation. Nevertheless, medical intervention is often required for significant defects. Traditional approaches for bone repair include external fixation methods such as bone grafting, the Ilizarov technique, and bone transport. However, some of these methods, particularly the Ilizarov technique, involve prolonged recovery periods and carry substantial risks. Bone grafts are generally classified into three categories: autografts, allografts, and xenografts [2]. Autologous bone grafts provide essential osteoinductive factors and osteogenic cells, offering superior outcomes in bone healing and regeneration. Despite these advantages, autografts are limited by inadequate tissue availability for large defects [3]. Metals and ceramics have been explored as potential alternatives for bone grafting. Metals provide immediate mechanical support but exhibit poor integration with host tissue and are susceptible to fatigue failure. Ceramics, while biocompatible, are brittle and have low tensile strength, making them unsuitable for regions subjected to torsional, bending, or shear stresses [4]. These limitations underscore the need for substitute materials that closely mimic the structural and functional properties of natural bone.

Tissue engineering (TE) represents a multidisciplinary approach that integrates principles of biology and engineering to restore, maintain, or improve tissue function. This emerging field addresses critical clinical challenges, including organ failure and tissue loss. Historically, organ transplantation, primarily allografts, was the predominant solution for damaged tissues. However, this approach is constrained by donor scarcity and the risk of immunological rejection. Autografts and xenografts have also been employed, though each presents unique limitations. Prosthetic devices offer another alternative but are associated with infection risks and poor long-term integration [5]. TE offers a promising solution by fabricating scaffolds from biocompatible materials to provide a structural matrix that supports cell growth and tissue formation. These scaffolds must mimic the natural extracellular environment, be nontoxic, and degrade safely over time [6].

The TE process involves three essential components: cells, scaffolds, and growth factors. Its applications include (i) providing cellular prostheses or replaceable parts to induce regeneration, (ii) creating tissue-like models for research, and (iii) developing delivery systems for therapeutic agents [6]. TE systems are classified into open and closed configurations. Open systems involve implanting cell-seeded scaffolds into the body, whereas closed systems use external devices to support organs. The standard TE workflow includes cell isolation via biopsy, in vitro expansion by seeding onto a scaffold, and implantation into the defect site. Scaffolds are typically three-dimensional, highly porous structures that serve as templates for cell regeneration. They are combined with growth factors and signaling molecules and maintained in bioreactors until a functional graft is formed. Advanced bioreactor systems are essential for controlling process parameters and creating optimal culture conditions [7].

**Figure 1:**
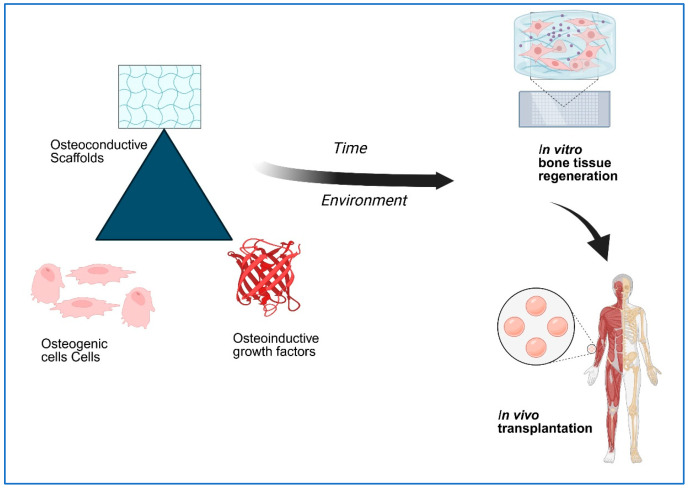
Schematic diagram of Bone Tissue Engineering Strategy (created with BioRender.com).

Bone TE offers a cost-effective, reliable, and physiologically compatible strategy for bone regeneration. TE has emerged as an alternative to conventional grafting procedures, which are often limited by donor tissue availability and procedural constraints [8]. Successful bone regeneration requires three key elements: osteoconductive scaffolds, osteogenic progenitor cells, and osteoinductive growth factors (Figure 1) [9]. Scaffolds act as temporary substrates that support cellular activity and maintain cell differentiation. The performance of scaffolds is critical to the success of bone TE. Biomaterials used in this context must exhibit properties such as osteoconductivity, biocompatibility, integration with surrounding tissue, and adequate mechanical strength. Integration is particularly important for enabling extracellular matrix (ECM) remodeling and facilitating physiological and biological changes within bone tissue [10].

Despite the rapidly expanding available literature on bone TE, a critical gap persists: the majority of existing reviews address scaffold design, cell biology, or growth factor delivery in isolation, without systematically examining how these elements interact across biological, mechanical, and fabrication dimensions to determine regenerative outcomes collectively. As a result, translational progress remains fragmented, and clinicians lack a unified framework for evaluating scaffold-based strategies in the context of real-world bone repair challenges. This review, therefore, aims to address a central question: How can the rational integration of osteoconductive scaffold architecture, osteogenic cellular mechanisms, and osteoinductive signaling be optimized to bridge the gap between laboratory advances and clinically viable bone regeneration strategies? By synthesizing current evidence across these intersecting domains, this review fills a gap in the literature by providing a comprehensive, cross-disciplinary analysis that moves beyond material-specific or cell-specific perspectives. Critically, it advances a conceptual framework that positions scaffold design not merely as a structural consideration, but as a dynamic, biologically instructive platform whose topographical, mechanical, and biochemical properties must be co-engineered to recapitulate the native bone microenvironment, a system whose fractal-like hierarchical organization, spanning the nanoscale to the macroscale, demands equally hierarchical design thinking in regenerative strategies [11,12]. This integrative perspective offers a new lens through which researchers and clinicians can evaluate emerging technologies, identify unresolved challenges, and prioritize future directions in bone TE.

This review highlights bone tissue engineering as a transformative strategy to address the inherent limitations of conventional grafting and fixation techniques. By integrating osteoconductive scaffolds, osteogenic cells, and osteoinductive growth factors, bone TE establishes a biologically inspired framework for effective regeneration. Recent advances in biomaterials, scaffold architecture, and fabrication technologies have markedly improved the ability to replicate native bone structure and function, yielding solutions that are both physiologically compatible and clinically viable. Despite persistent challenges in achieving complete integration and long-term stability, ongoing research continues to optimize scaffold performance and cellular approaches, positioning TE at the forefront of regenerative medicine and as a compelling alternative to traditional bone repair modalities.

## 2. Natural Bone Structure and Composition

Bone is a hierarchical composite material integrating organic and inorganic phases to achieve exceptional mechanical performance and adaptability. The organic matrix, primarily type I collagen, provides tensile strength and toughness, while embedded carbonated-substituted hydroxyapatite (HA) crystals confer compressive stiffness and rigidity [13]. At the nanoscale, bone comprises mineralized collagen fibrils, in which rod- or plate-like crystallites (∼5 × 5 × 50 nm) are embedded within the collagen matrix [13,14]. The organized mineralization forms concentric lamellae in osteons, centered around Haversian canals and connected by Volkmann’s canals, facilitating vascular and nutrient transport [15]. Quantitative composition analysis indicates bone tissue consists of approximately 30% organic matrix, 60% mineral, and 10% water by weight (Table 1) [16]. At the macroscopic level, bone architecture diverges into two structural types: (i) Cortical bone: Dense and load-bearing, characterized by ~10% porosity, an elastic modulus of 15–20 GPa, and compressive strength of 100–200 MPa [17]. (ii) Cancellous bone: Spongy and metabolically active, with 50–90% porosity, modulus spanning 0.1–2 GPa, and compressive strength of 2–20 MPa [17,18].

Bone’s hierarchical structure from nano- to macro-scale forms a mechanically efficient framework: mineral lamellae and collagen fibrils at the nanoscale; osteons and interstitial lamellae at the microscale; and coordinated cortical and trabecular networks at the macroscale [11,15].

### 2.1. Bone Cells and Their Functions

**Figure 2:**
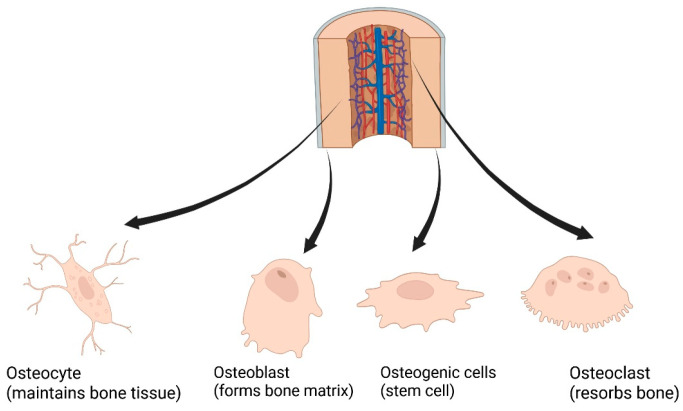
Schematic diagram of different types of bone cells (created with BioRender.com).

Bone remodeling and repair depend on the coordinated activity of four distinct cell types:

(i) Osteoprogenitor cells are multipotent mesenchymal precursors residing in periosteal and endosteal niches; they differentiate into osteoblasts, chondrocytes, adipocytes, fibroblasts, or myoblasts during growth and regeneration [19].
(ii) Osteoblasts secrete the organic bone matrix and regulate mineralization; once encapsulated, they become osteocytes. They also modulate osteoclast activity via paracrine signaling [19].
(iii) Osteocytes, the mechanosensory cells embedded within the matrix, maintain mineral homeostasis and orchestrate bone adaptation in response to mechanical cues [20].
(iv) Osteoclasts are large, multinucleated cells that enzymatically resorb bone during remodeling and healing, critical for maintaining structural integrity [19]. Figure 2 shows the schematic representation of different types of bone cells.

### 2.2. Bone Healing Process

Bone repair occurs through three overlapping phases [21,22]. In the inflammatory phase, hematoma formation and immune cell infiltration trigger angiogenesis and recruit mesenchymal precursors [21]. During the repair phase, a soft callus of fibrous tissue and cartilage develops, which gradually transitions into woven bone as osteoblasts deposit mineralized matrix [22]. Finally, in the remodeling phase, the immature woven callus is replaced by organized lamellar bone; osteoclasts resorb the initial bone while osteoblasts restore structural integrity [22].

### 2.3. Mechanical Properties of Natural Bone

The mechanical behavior of bone is primarily assessed in vivo, while its mechanical properties are typically determined in vitro. These tests follow standardized protocols originally designed for conventional materials such as metals and plastics. On a smaller scale, composite models can be used to evaluate mechanical performance [11,13]. Brittle apatite contributes to bone’s stiffness, whereas collagen provides a tough, flexible matrix. The interaction between these two components significantly influences the bone’s tensile properties. Understanding bone structure is essential for replicating its characteristics in implant materials. Cancellous bone consists of an interconnected network of plates and rods, with density ranging from 0.05 to 0.7 g/cm^3^ [18]. Compression tests reveal that cancellous bone exhibits characteristics typical of cellular solids.

The outer region of bone, known as cortical bone, forms a dense cylindrical shell along the bone’s length. It has approximately 10% porosity, a modulus of 50–150 MPa, and a compressive strength of 12–18 MPa [17]. In contrast, cancellous bone, located at the distal ends, has a spongy structure with about 75% porosity, a modulus ranging from 10–500 MPa, and compressive strength between 10–20 MPa [17,18].

Overall, cortical bone is denser and harder than cancellous bone, which is comparatively softer and less dense. Both types contain a collagen matrix embedded with mineral crystals, providing structural support and mechanical strength. An increase in bone density corresponds to thicker cell walls, which enhance the compressive modulus and raise the plateau stress while reducing strain at the onset of densification. Bone also responds dynamically to mechanical loading; physical stress influences bone formation and resorption. Designing scaffolds with mechanical properties similar to natural bone is critical for supporting injured areas. Material selection affects key characteristics, including porosity, formation, and overall mechanical performance. Bone exhibits anisotropy and plasticity under loading, influenced by its mineral–matrix ratio. Studies show an optimal composition window that maximizes stiffness, strength, and toughness, guiding bone-mimicking material design [16].

### 2.4. Properties of Bone TE Scaffold

Recent advances in scaffold technology aim to address limitations associated with organ transplantation and bone failure treatments. Scaffolds are designed to influence the chemical, physical, and biological environment surrounding cell populations. An optimized scaffold must integrate both biological and mechanical characteristics of bone tissue. The biological environment of bone involves fluid and nutrient transport as well as the presence of various cell types. A scaffold tailored to these conditions can effectively stimulate cell migration and extracellular matrix deposition [23,24]. Conversely, the mechanical environment encompasses loading requirements and spatial organization of bone cells to promote cell–cell signaling. Scaffolds engineered for these conditions can successfully transfer mechanical loads [25,26].

The primary goal of scaffold design is to fabricate an ideal matrix structure. Scaffold architecture plays a critical role in creating a biocompatible environment for osteogenic cells [27]. Bone scaffolds enable cells to generate tissue with specific shape, size, and functionality, making scaffold design and fabrication central to biomaterials research and regenerative medicine [28]. Scaffold performance depends on several design variables, including microstructure, pore size, porosity, mechanical properties, and surface chemistry. The microstructure should mimic native bone tissue, incorporating extracellular matrix-like features. Adequate porosity and interconnected pores are essential for nutrient and waste transport. In vitro diffusion is limited to 100–300 μm; therefore, scaffolds must ensure that cells reside within 300 μm of capillary networks [29].

#### 2.4.1. Chemical and Mechanical Properties of Bone TE Scaffold

Studies have shown that bone defects can be effectively repaired using scaffolds seeded with appropriate cell densities or embedded with growth factors [30]. For successful osseous tissue regeneration, the scaffold must exhibit four key chemical properties: osteoconductivity, which ensures a porous structure that supports cell attachment, migration, and nutrient exchange; osteoinductivity, which involves the incorporation of growth factors to induce mesenchymal stem cell differentiation into osteoblasts; osteogenicity, which promotes osteoblast activity for mineral deposition and collagen calcification; and osteointegration, which enables the newly formed mineralized tissue to establish strong bonding with the surrounding implant site [30].

**Figure 3:**
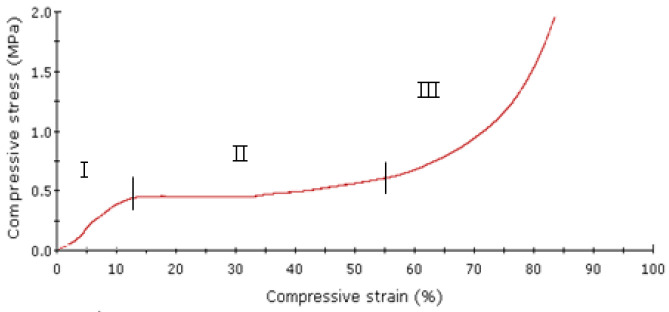
Typical compressive curve of the polymer scaffolds. Region I: Linear elasticity, region II: plateau, region III: densification.

Figure 3 shows a typical stress–strain behavior of a polymer scaffold. Both polymer scaffolds and wet cancellous bone exhibit compressive stress–strain curves with three regions: an initial linear elastic phase, a plateau region associated with structural collapse, and a densification phase in which stress rises sharply. However, cancellous bone demonstrates significantly higher stiffness and strength due to its mineralized trabecular architecture, with mechanical properties strongly dependent on relative density; as density increases, elastic modulus and yield stress scale approximately with the square of density, following Gibson–Ashby models. In contrast, highly porous polymer scaffolds exhibit lower modulus and plateau stress, with porosity playing a dominant role in reducing load-bearing capacity. However, increased porosity in both systems enhances permeability. However, it compromises mechanical integrity, making cancellous bone inherently superior for load-bearing applications, while PHBV scaffolds prioritize biocompatibility and tissue ingrowth over structural strength.

#### 2.4.2. Physical Properties of Bone TE Scaffold

An ideal bone scaffold should mimic natural bone structure and exhibit characteristics such as 3D architecture, appropriate porosity, interconnected pores, and optimal pore size [29]. Osteoconductivity and pore architecture significantly influence osteoprogenitor cell colonization [31]. Precise pore design is critical for effective matrix structure [31].

#### 2.4.3. Surface Properties of Bone TE Scaffold

Surface properties play a critical role in scaffold interaction with host tissue. Surface roughness influences osteoblast function and overall outcomes [31]. AFM imaging reveals collagen fibril networks with 67 nm periodicity [32]. Surface modification strategies include coatings, plasma treatment, and incorporation of bioactive compounds [33,34]. The interaction zone between tissue and implant is ~0.1–1 nm [35]. Collagen or conductive polymer coatings enhance hydrophilicity and cell adhesion [36,37,38]. Therefore, surface engineering is a key consideration in tissue engineering applications.

Khan et al. (2008) [30] demonstrated that variations in surface roughness and pore size significantly affect cellular behavior. Furthermore, nanoscale surface modifications can enhance biological performance. For example, HA/PHBV composite scaffolds exhibit a fibrous morphology with a higher surface-to-volume ratio than scaffolds with solid pore walls. Such structures improve protein adsorption and facilitate cell attachment and proliferation, thereby enhancing scaffold functionality [30]. Gas plasma treatment is one promising method for introducing functional groups onto polymer surfaces through covalent modification. Biochemical surface modifications can further modulate tissue responses. For instance, collagen, a primary component of bone matrix, can be used to coat scaffold surfaces. Collagen contains specific amino acid sequences that bind directly to cell-surface receptors, improving cell adhesion. Yang et al. (2002) reported that coating poly-D, L-lactide (PDLLA) with collagen enhanced hydrophilicity and surface roughness, resulting in improved cell activity [37]. Similarly, conductive PEDOT-PSS coating on PHBV/PLA electrospun fibers significantly increased hydrophilicity and promoted cell attachment and proliferation [38].

#### 2.4.4. Bioactivity Properties

Bioactivity remains a cornerstone in scaffold design, as it determines the material’s ability to bond with living tissue under physiological-like conditions. Recent studies have reinforced the importance of simulated body fluid (SBF) testing as a predictive tool for in vivo performance. Shendage et al. [39] demonstrated that cellulose-based scaffolds impregnated with calcium silicate and turmeric exhibited hydroxyapatite formation during SBF immersion, alongside hemocompatibility and enhanced osteogenesis in vivo. Similarly, TiO_2_ scaffolds pre-treated in 10× SBF developed a biomimetic apatite layer, significantly improving bone cell adhesion and proliferation [40]. These findings highlight that SBF-induced apatite deposition not only validates scaffold bioactivity but also serves as a critical step toward optimizing osteointegration and regenerative potential.

Current studies demonstrate that incorporating conductive polymers into scaffolds enhances osteogenic differentiation and mineralization, particularly when combined with electrical stimulation [41,42]. Polypyrrole (PPy) thin films prepared on tricalcium phosphate (TCP) substrates via admicellar polymerization have also been investigated for their influence on mesenchymal stem cells (MSCs). Castano et al. reported that PPy film thickness, regulated by pyrrole monomer concentration, significantly affected MSC adhesion, proliferation, and alkaline phosphatase activity, with optimal adhesion observed at 20 × 10^−3^ M [43]. Higher monomer concentrations reduced cell attachment and calcified matrix formation. Bendrea et al. later confirmed that these effects were attributable to surface morphology rather than cytotoxicity [44]. More recent work has expanded on these findings, showing that polymer films such as P3HT and PPy derivatives promote osteoblastic differentiation of MSCs and adipose-derived stem cells, underscoring their promise in regenerative medicine [45,46]. Recent advancements have refined these models by incorporating age- and sex-specific calcium kinetics [47] and linking calcium fluxes to bone metabolism and energy regulation [48]. These updated frameworks enhance our understanding of calcium dynamics in both nutritional and orthopedic contexts.

#### 2.4.5. Degradation Properties

The hydrolytic degradation rate of polymeric scaffolds is a critical parameter in biomedical applications. Degradation behavior directly influences implant performance, drug release kinetics, and the mechanical stability of scaffolds over time. The primary motivation for using absorbable and biodegradable polymers is to avoid secondary surgical procedures by ensuring complete dissolution of implant components or by providing time-dependent mechanical properties. Furthermore, controlled degradation enables the release of bioactive agents such as growth factors or antibiotics from polymer matrices. Both material-related and medium-related factors strongly affect degradation kinetics. Key material factors include molecular structure (molecular weight and distribution, comonomer composition, terminal groups, crosslink density, tacticity, and branching), morphology (shape, dimensions, pore size, porosity), and highly ordered structures (crystallinity, spherulitic size, and orientation). Medium-related factors include pH, temperature, solutes, microbial activity, enzyme activity, and mechanical stress. Among these, molecular weight, crystallinity, porosity, pH, and temperature are particularly influential. For example, the presence of catalytic molecules, incorporation of hydrophilic monomers, and variations in pH or temperature significantly alter degradation rates. Increased molecular weight and crystallinity generally reduce hydrolytic degradation.

Polymer degradation can proceed via surface erosion or bulk erosion [49]. In surface erosion, only the outer layers are degraded by ions or catalytic molecules, leaving the scaffold core intact. This mechanism is typically observed when enzymes or alkalis are present in the degradation medium. In contrast, bulk erosion occurs in the absence of catalytic molecules, such as in phosphate-buffered solutions, where water penetrates the polymer matrix, and hydrolysis occurs throughout. Material thickness is a critical determinant of mechanism. Crystalline polymers, characterized by numerous spherulites, degrade preferentially in their amorphous regions, as these regions contain chains that are more susceptible to degradation. Early degradation often involves surface erosion, followed by bulk erosion as molecular weight decreases and soluble oligomers form. Carboxyl end groups further catalyze chain cleavage, accelerating degradation in amorphous regions [50]. Major factors that affect polymer degradation are polymer morphology, composition, molecular weight, additives, specimen size, and porosity [51].

#### 2.4.6. Scaffold Properties: Physicochemical Characteristics and Functional Outcomes

The physicochemical properties of scaffolds are active determinants of biological performance and clinical success rather than merely required parameters. Porosity and pore interconnectivity directly regulate nutrient diffusion, vascular ingrowth, and metabolic waste removal, processes critical for scaffold survival beyond the diffusion limit of approximately 200 µm in avascular constructs. Pore sizes of 100–400 µm support osteoblast migration and bone ingrowth, while interconnected macroporosity above 300 µm promotes vascularization, which is essential for load-bearing applications. However, increasing porosity to enhance biological performance inevitably compromises mechanical integrity. This fundamental design trade-off remains inadequately resolved for load-bearing sites and is frequently underreported in the literature [11,12]. Surface topography, stiffness, and chemistry operate synergistically to modulate cell adhesion, mechanotransduction, and osteogenic differentiation. Micro- and nanotopographical features promote integrin binding, cell adhesion, and activation of osteogenic signaling cascades, translating into measurable increases in alkaline phosphatase activity, collagen type I deposition, and mineralization. Scaffold stiffness matching native osteoid (~25–40 kPa) directs MSC differentiation toward osteogenic lineages through matrix mechanosensing. At the same time, surface chemistry governs protein adsorption patterns that precede and mediate cell attachment, hydrophilic surfaces preferentially adsorbing fibronectin and vitronectin in conformations that expose integrin-binding Arginine-glycine-aspartic (RGD) domains [13]. Collectively, these properties must be treated as an integrated design space in which trade-offs between mechanical performance, biological activity, degradation rate, and manufacturability are systematically balanced to produce scaffolds that are not only effective in the laboratory but also scalable, reproducible, and clinically deployable.

### 2.5. Bone-Substituted Biomaterials

When a biomaterial is exposed to body fluids, it interacts directly with internal tissues, imposing strict requirements on its properties. The foremost criterion is biocompatibility; the material must not provoke adverse biological responses and remain stable within the body until new tissue forms. Additionally, it must be nontoxic and noncarcinogenic, which limits the range of suitable engineering materials. Beyond biological compatibility, the biomaterial should exhibit adequate physical and mechanical properties to function as a replacement or augmentation for body tissues. Practical considerations include ease of fabrication, cost-effectiveness, and availability. Broadly, biomaterials fall into three categories: metals, ceramics, and polymers.

Material selection for scaffolds depends on biocompatibility, biodegradability, mechanical properties, and cellular interactions. Surface biocompatibility is influenced by surface chemistry, which affects the adsorption of biological molecules and regulates cell function. Other factors include molecular weight, solubility, shape, hydrophobicity/hydrophilicity, surface energy, and water absorption. For bone tissue engineering, commonly used materials include metals, bioceramics, and biopolymers.

#### 2.5.1. Metals

Currently, commonly used orthopedic metals include: stainless steels (F55, F138, 316L), cobalt–chromium alloys (F75, F90), and titanium and its alloys (F67, commercially pure titanium; F136, Ti-6Al-4V). Stainless steel is widely used for fracture fixation due to its high tensile strength, ductility, and ability to be cold-worked—an essential feature for shaping plates during surgery. However, its use in artificial joints declined because early castings lacked adequate fatigue resistance. Fatigue properties are critical since an artificial hip joint experiences millions of load cycles annually, with forces reaching two to three times body weight during walking. Titanium alloys, such as Ti-6Al-4V, are preferred for joint prostheses because their elastic modulus is roughly half that of stainless steel and cobalt–chromium alloys, reducing the risk of stress shielding [52].

In addition to these permanent metals, biodegradable metals such as magnesium, zinc, and iron alloys have recently emerged as promising alternatives for temporary orthopedic implants. Table 2 shows a comparative overview of permanent and biodegradable metals for orthopedic applications. These materials provide mechanical support during the critical healing phase and gradually degrade in vivo, eliminating the need for secondary surgeries to remove hardware. Magnesium alloys, in particular, have been extensively studied for fracture fixation due to their favorable biocompatibility and mechanical properties, though challenges remain in controlling their corrosion rate [53]. Zinc-iron alloys are also under investigation, offering slower degradation profiles and potential applications in load-bearing implants [54]. Collectively, biodegradable metals represent a new frontier in orthopedic biomaterials, combining structural performance with resorbability to reduce patient morbidity and healthcare costs [55]. The emergence of biodegradable metals offers a promising alternative, eliminating the need for secondary removal surgeries and enabling gradual load transfer to healing tissues. Ongoing research continues to optimize their mechanical properties and biocompatibility for broader clinical use.

#### 2.5.2. Bioceramics

Bioceramics have long been used in dentistry, but their orthopedic applications are mainly limited to repairing or replacing hard tissues, such as bone. Bone itself is a composite of organic material and a ceramic phase dominated by calcium hydroxyapatite (HA), which has a Ca/P ratio of about 1.67. Synthetic HA is widely used because it bonds well with natural bone and is highly biocompatible, bioactive, and osteoconductive. It is commonly applied as a coating on metal implants to improve long-term fixation. However, pure HA is too brittle and weak for load-bearing uses. To address these mechanical limitations, HA is often combined with polymers or biopolymers to form composite scaffolds, such as PHBV/HA and PCL/HA systems, thereby improving stability and supporting osteogenic activity [58,59]. Maintaining an ideal Ca/P ratio (1.50–1.67) is essential for synthetic HA to support bone regeneration.

Nano-hydroxyapatite (nHA), which mimics the nanostructure of natural bone mineral, offers enhanced surface area, protein interaction, and bioactivity. Compared to micro-scale HA, nHA promotes better cell adhesion and calcium deposition. Multiple synthesis methods exist for producing micro- and nanoscale HA, including emulsion, co-precipitation, sol–gel, and microwave techniques. Table 3 represents a comparative overview of micro- vs. nano-hydroxyapatite in TE applications.

Other Ceramic Materials in Orthopedic Tissue Engineering include bioinert ceramics such as alumina (Al_2_O_3_) and zirconia (ZrO_2_), which are widely used in load-bearing implants due to their high hardness, wear resistance, and chemical stability. Alumina has been applied in femoral heads and dental prostheses, while zirconia offers superior fracture toughness and is increasingly used in hip replacement acetabular cups and dental implants [62]. Bioactive glasses and glass-ceramics (e.g., 45S5 Bioglass^®^) exhibit osteoconductivity and osteoinductivity by forming a hydroxycarbonate apatite layer that bonds directly with bone. These materials are employed in bone fillers, scaffolds, and coatings for metallic implants, with glass-ceramics providing improved mechanical strength compared to pure bioactive glass [62]. Calcium phosphate ceramics beyond HA, such as tricalcium phosphate (TCP) and biphasic calcium phosphate (BCP), are resorbable and support bone regeneration. TCP degrades faster than HA, making it suitable for temporary scaffolds, while BCP combines HA and TCP to balance bioactivity with controlled resorption [57].

Composite and porous ceramics have also gained attention, particularly for scaffolds designed with interconnected pores to facilitate vascularization and nutrient exchange. Ceramic-polymer composites, such as HA combined with biodegradable polymers (PLA, PCL), overcome brittleness and enhance mechanical properties while maintaining osteogenic potential [63]. Overall, ceramic biomaterials provide a versatile toolkit for orthopedic tissue engineering. Their spectrum of properties, from bioinert stability to bioactive bonding and controlled biodegradability, enables tailored solutions for bone regeneration, implant coatings, and scaffold design.

#### 2.5.3. Polymers

Polymers are classified as natural or synthetic biopolymers. Natural and synthetic biopolymers each offer distinct advantages and limitations in tissue engineering. Natural polymers (collagen, gelatin, chitosan) provide excellent biocompatibility and bioactivity, making them ideal for promoting cellular functions. However, their mechanical weaknesses limit their use in load-bearing applications. In contrast, synthetic polymers (PLA, PLGA, PCL, etc.) offer superior mechanical strength, predictable degradation profiles, and scalable production, but often lack the biological cues necessary for optimal cell interaction. To overcome these individual shortcomings, hybrid scaffolds that integrate both natural and synthetic polymers are increasingly being developed. These composite systems aim to combine the biological functionality of natural materials with the structural integrity of synthetic materials, representing a promising direction for advancing clinically effective tissue engineering solutions [64,65].

Recently, conductive polymers (CPs) have emerged as a critical class of materials in TE due to their unique ability to mediate electrical stimulation, a key factor in regulating cellular behavior. Their conjugated structures enable electronic conductivity, making them suitable for applications in neural interfaces, cardiac patches, biosensors, TE scaffolds, and drug delivery systems. Among the most studied CPs are polypyrrole (PPy), polyaniline (PANI), poly(3,4-ethylenedioxythiophene) (PEDOT), and its doped form, PEDOT: PSS. Importantly, electrical stimulation has been shown to enhance osteoblast proliferation, adhesion, and gene expression, underscoring the therapeutic potential of CP-based scaffolds [64]. While challenges remain, particularly stability and degradation in biological environments, composite systems that blend CPs with biodegradable polymers offer promising solutions, combining electrical functionality with improved biocompatibility and mechanical strength. Their unique combination of biocompatibility, tunable conductivity, and versatility in scaffold design positions them as a critical bridge between traditional biomaterials and next-generation bioelectronic interfaces, paving the way for advanced regenerative therapies and functional tissue restoration.

### 2.6. Fabrication Techniques of Tissue Engineering Scaffolds

**Figure 4:**
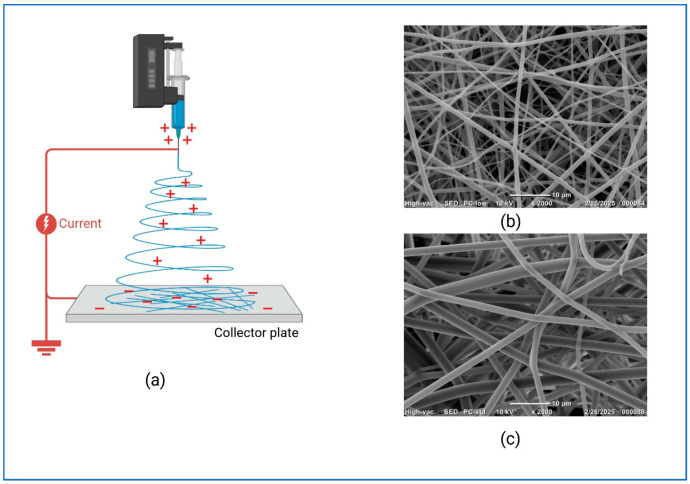
Polymer scaffold fabrication using electrospinning. (**a**) Electrospinning set-up; (**b**,**c**) scanning electron microscope of scaffolds using different needle sizes.

Various fabrication techniques have been developed to produce scaffolds for tissue engineering. These methods aim to produce biodegradable, bioresorbable polymeric scaffolds with high porosity and surface area. Each technique offers distinct advantages and limitations. The most commonly used techniques are gas foaming, rapid prototyping (e.g., 3D or 4D printing), selective laser sintering, electrospinning, and freeze drying [64]. Among the techniques, electrospinning is an effective method for producing nanofibrous scaffolds that replicate the extracellular matrix, providing a biomimetic environment for tissue engineering. Figure 4 shows the SEM analysis of electrospun PVP scaffolds with varying fiber diameters, highlighting the sensitivity of electrospinning to parameter changes. The results emphasize the need for precise optimization to produce scaffolds using conventional techniques with uniform morphology, high porosity, and an interconnected architecture, which are essential for cell adhesion, nutrient transport, and tissue integration. Table 4 and Table 5 compare various scaffold fabrication techniques and provide a summary of scaffold classes, manufacturing methods, and translational limitations in bone tissue engineering.

## 3. In Vitro Biological Evaluation of Bone Tissue Engineering Scaffolds

In vitro biological evaluation plays a critical role in assessing the biocompatibility, osteogenic capacity, and overall functional performance of bone tissue engineering scaffolds prior to in vivo experimentation. These studies provide essential insights into cell–scaffold interactions, degradation kinetics, and scaffolds’ ability to support osteogenic differentiation under controlled laboratory conditions. Cytocompatibility is commonly examined through assays such as MTT, Live/Dead staining, and Alamar Blue, with recent work on nano-carbonated hydroxyapatite/chitosan scaffolds reporting over 90% cell viability and improved osteoblast adhesion [66]. Osteogenic differentiation is typically evaluated by monitoring alkaline phosphatase activity, osteocalcin production, and RUNX2 expression, and oxygen-releasing PLA/CPO scaffolds have been shown to significantly enhance these markers under dynamic culture conditions [67]. To further mimic the physiological microenvironment, co-culture systems incorporating osteoblasts and endothelial cells are increasingly employed, with composite hydrogel scaffolds demonstrating enhanced angiogenic signaling and vascularization potential [68]. Scaffold morphology, particularly pore size and interconnectivity, also plays a decisive role in nutrient diffusion and cell migration; for example, PLA scaffolds engineered with 0.6 mm pores have shown improved cell infiltration and mechanical stability [69]. Dynamic culture systems, including bioreactors, provide mechanical stimulation and improved nutrient exchange, and biomimetic dual-sensing scaffolds have exhibited superior mineral deposition under these conditions [70]. To ensure consistency and reproducibility across studies, standardized guidelines, such as ASTM F2739, outline validated methods for quantifying cell viability and related biological responses in biomaterial scaffolds [71].

## 4. In Vivo Biological Evaluation of Bone Tissue Engineering Scaffolds

In vivo evaluation of bone tissue engineering scaffolds often employs rabbit radial defects, rat calvarial defects, and canine mandibular models to monitor bone regeneration, vascularization, and mechanical integrity longitudinally. Common assessment methods include micro-computed tomography (micro-CT) for quantifying new bone volume, histological staining to examine tissue integration, and gene expression analysis to detect activation of osteogenic and angiogenic markers. While these models have yielded valuable mechanistic insights, their limited biomechanical complexity and their immunological relevance to human physiology represent a critical, frequently underacknowledged translational barrier, as discussed further below.

One notable advancement is the development of prevascularized, mineralized nanofibrous scaffolds composed of polylactic acid (PLA), hydroxyapatite, and decellularized vascular tissue. When implanted in rabbit radial defects, these scaffolds exhibited bone formation similar to that of allografts within 8 weeks, with micro-CT confirming robust neovascularization [72]. Additionally, 3D-printed magnesium-silicate/β-tricalcium phosphate (MS/β-TCP) scaffolds tested in rat calvarial defects achieved controlled release of Mg^2+^ and Si^4+^ ions, activating the PI3K/Akt signaling pathway and significantly enhancing both bone volume and vessel density over 6–12 weeks [73]. In intraoral applications, electrospun scaffolds coated with the AMP2 peptide were evaluated in beagle mandibular defects. They demonstrated guided bone regeneration comparable to that achieved with xenografts, without the need for barrier membranes, simplifying the surgical procedure while maintaining osteogenic efficacy [74]. For infected bone defects, a 3D-printed titanium alloy scaffold featuring a triply periodic minimal surface and sequential dual delivery of BMP-2 and antibiotic-loaded PLGA microspheres demonstrated effective eradication of infection. It promoted bone healing in rat models [75]. Composite PLA–calcium phosphate glass scaffolds further enhanced angiogenesis and vascular maturation in mouse subcutaneous implantation models, with mature vascular networks observed by week four, underscoring the importance of scaffold architecture in vascular integration [76].

Collectively, these studies highlight several emerging trends: early angiogenesis is essential for scaffold integration and long-term bone repair; bioactive ionic release such as Mg^2+^ and Si^4+^ can concurrently stimulate osteogenesis and angiogenesis; multifunctional scaffolds combining osteoinductive and antimicrobial agents are effective against infected bone defects; and architectural optimization, including hierarchical porosity, prevascularization, and controlled drug delivery, remains key to replicating native bone microenvironments and enhancing healing outcomes.

Despite these promising in vivo findings, significant barriers continue to impede the translation of bone TE scaffolds from the laboratory to clinical practice, and these challenges deserve more rigorous and transparent discussion than they typically receive in the literature. The regulatory pathway for complex, bioactive scaffolds combining cells, biomaterials, and therapeutic agents is evolving but remains inconsistent across jurisdictions. In the United States, such constructs may be classified as combination products subject to overlapping FDA oversight, while in Europe, they fall under the Advanced Therapy Medicinal Products (ATMP) framework, both pathways involving lengthy, costly, and uncertain approval timelines that discourage commercial investment. Bridging the gap between laboratory innovation and clinical deployment will ultimately require not only scientific optimization but sustained collaboration among material scientists, clinicians, regulatory bodies, and industry partners to establish standardized preclinical testing protocols, harmonized regulatory frameworks, and scalable manufacturing pipelines that can deliver safe, effective, and accessible bone TE solutions to patients with complex skeletal defects.

## 5. Clinical Translation Challenges

Despite promising preclinical outcomes, translating bone tissue-engineering scaffolds into clinical practice poses numerous obstacles. Technical challenges persist in scaling up production of complex architectures—such as hierarchical porosity and patient-specific designs—to Good Manufacturing Practice (GMP) standards without compromising scaffold integrity or reproducibility [77]. Regulatory hurdles further complicate clinical adoption: multifunctional scaffolds combining osteoinductive, antimicrobial, or angiogenic components often blur the lines between medical devices, biologics, and combination products, requiring extensive safety and efficacy data for approval. Additionally, managing the host immune response to implanted scaffolds is critical; evidence shows that residual decellularization agents or scaffold degradation products can shift macrophage polarization toward a pro-inflammatory state, impairing integration [78].

Challenges inherent to 3D printing technologies—including material selection, print resolution, and control of cell–material interactions—remain significant barriers to clinical use. As noted by Zhao et al. (2024), discrepancies between lab-scale successes and clinical outcomes underscore the need for standardized protocols and translational frameworks [79]. Finally, commercialization is hindered by high production costs, limited reimbursement pathways, and a lack of long-term human data demonstrating clinical benefit and cost-effectiveness. Overcoming these challenges requires a coordinated approach involving robust manufacturing workflows, interdisciplinary collaboration, and early engagement with regulatory and funding agencies [77].

## 6. Limitations and Future Directions

Despite significant advancements in scaffold design and in vivo validation, several gaps remain in translating these technologies into clinical practice. First, most studies rely on small-animal models that do not fully replicate the biomechanical and immunological complexity of human bone healing. Large-animal models and long-term studies are needed to evaluate scaffold performance under load-bearing conditions and in chronic disease states such as osteoporosis or diabetes. Second, while angiogenesis and osteogenesis have been widely studied, the immune response to scaffolds, particularly the roles of macrophage polarization and inflammatory signaling, warrants deeper investigation to ensure predictable outcomes across diverse patient populations. Another critical gap lies in integrating multifunctional properties. Although scaffolds with combined osteoinductive and antimicrobial capabilities have shown promise, their long-term stability, controlled release kinetics, and potential cytotoxicity remain poorly understood. Furthermore, current fabrication techniques, including 3D printing and electrospinning, offer excellent architectural control but often lack scalability and cost-effectiveness for clinical translation. Finally, regulatory pathways for complex, bioactive scaffolds are still evolving, creating uncertainty in commercialization timelines.

A critical evaluation of scaffold design must move beyond merely cataloging materials and fabrication approaches toward a mechanistic understanding of how specific scaffold features govern key biological processes. Scaffold topography, stiffness, porosity, and surface chemistry are not passive structural attributes; rather, they actively modulate cell adhesion and mechanotransduction by providing physical and biochemical cues that cells sense and translate into intracellular signaling cascades [11,12]. For instance, substrates with stiffness matching that of native bone (~25–40 kPa for osteoid to >40 kPa for mineralized bone) have been shown to direct mesenchymal stem cell (MSC) differentiation toward osteogenic lineages. At the same time, scaffold microarchitecture influences focal adhesion kinase (FAK) activation and cytoskeletal organization, both of which are critical mediators of osteogenic commitment. Similarly, pore geometry and interconnectivity directly regulate nutrient diffusion, vascularization, and ultimately ECM remodeling, processes that determine whether a scaffold supports transient repair or long-term functional regeneration. Despite the breadth of materials investigated, including bioceramics, natural and synthetic polymers, and their composites, existing studies frequently report these platforms as promising without rigorously comparing their relative performance under standardized conditions or transparently reporting their limitations. For example, while hydroxyapatite-based scaffolds offer excellent osteoconductivity, their brittleness under cyclic loading remains an unresolved limitation that is often understated in the literature. Likewise, while polymer-ceramic composites improve mechanical versatility, their degradation kinetics and byproduct toxicity require more systematic comparative evaluation. The integrative framework proposed by Bini et al. [78] explicitly linked native tissue architecture, biomimetic design principles, and biofabrication strategies in the context of osteochondral regeneration, demonstrating that effective regenerative approaches must be grounded in a structured understanding of how tissue organization informs scaffold design.

Future research should focus on developing smart scaffolds that respond to local biological cues, incorporating biodegradable sensors for real-time monitoring of healing, and leveraging personalized medicine approaches, such as patient-specific scaffold designs informed by advanced imaging and computational modeling. Additionally, combining scaffold technology with stem cell therapy, gene delivery, and growth factor gradients could further enhance regenerative outcomes. Collaborative efforts between material scientists, clinicians, and regulatory bodies will be essential to bridge the gap between laboratory innovation and clinical application.

## 7. Conclusions

Bone tissue engineering has emerged as a transformative alternative to conventional repair strategies, directly addressing the limitations of autografts, allografts, and xenografts, as well as metallic or ceramic implants, including donor scarcity, immunogenicity, brittleness, and poor long-term integration. This review has synthesized the foundational principles of bone TE with particular emphasis on scaffold design as the central determinant of regenerative success. Critically, scaffolds must be understood not as passive structural templates but as biologically instructive platforms whose topography, stiffness, porosity, and surface chemistry collectively govern cell adhesion, mechanotransduction, osteogenic differentiation, and ECM remodeling, a mechanistic perspective essential for advancing the field beyond descriptive material inventories toward rationally designed, outcome-driven regenerative systems.

## Figures and Tables

**Table 1:** Bone Composition.

Component	Site or Specific Molecule	Volume (%)
Water	Free/pore; bound at collagen-mineral interfaces; structural	15–25 vol %
Organic matrix	Type I collagen (~90 wt %), non-collagenous proteins (~10 wt %)	~32–40 vol %
Mineral (apatite)	Hydroxyapatite with carbonate substitutions	35–45 vol %

**Table 2:** Comparative Overview of Permanent vs. Biodegradable Metals in Orthopedic Applications.

Category	Examples	Key Properties	Biomedical Applications	References
Permanent Metals	Stainless steel, Titanium alloys (Ti-6Al-4V), cobalt–chromium alloys	High mechanical strength, corrosion resistance, long-term stability, and non-degradable	Joint replacements, fracture fixation devices, spinal implants	[56]
Biodegradable Metals	Magnesium alloys, Zinc alloys, Iron-based alloys	Biodegradable in a physiological environment, good biocompatibility, and promotes bone growth.	Temporary fracture fixation, bone scaffolds, pediatric implants	[57]

**Table 3:** Comparative Overview of Micro- vs. Nano-Hydroxyapatite in TE Applications.

Form of HA	Key Properties	Biomedical Performance	References
Micro-hydroxyapatite (Micro-HA)	Lower surface area, larger particle size (1–100 µm), lower reactivity, limited mechanical strength	Moderate osteoconductivity, slower resorption, less effective in promoting cell adhesion and proliferation	[60]
Nano-hydroxyapatite (Nano-HA)	High surface area, nanoscale particle size (<100 nm), enhanced reactivity, better mechanical integration	Superior osteoconductivity, faster resorption, improved cell adhesion, proliferation, and differentiation	[61]

**Table 4:** Comparison of Scaffold Fabrication Techniques and their advantages and limitations.

Technique	Description	Advantages	Limitations
Gas Foaming	Uses blowing agents (CO_2_, N_2_) under supercritical conditions to create porous polymer structures.	Solvent-free process; low temperature reduces polymer degradation.	Produces small pore sizes; requires ultrasound for better interconnectivity.
Sintering	Compacts ceramic powders using heat or pressure without melting.	Allows control over porosity; suitable for ceramics.	High fragility; poor pore interconnectivity.
Electrospinning	Uses an electric field to produce nanofibers from polymer solutions.	Mimics ECM structure; versatile for different polymers.	Limited control over 3D architecture; requires post-processing.
Casting & Particle Leaching	Introduces porogens (salt, sugar) into the polymer solution; leached to form pores.	Produces highly porous scaffolds; simple method.	Time-consuming; residual particles may remain.
Polymer Phase Separation (TIPS)	Thermally induced phase separation to control pore morphology.	Effective for micro- and nanoscale pores; adaptable for drug delivery.	Requires precise control of parameters; solvent handling needed.
Rapid prototyping (3D printing)	CAD-based layer-by-layer fabrication for complex architectures.	High precision; customizable for patient-specific defects.	Expensive equipment; slower than conventional methods.
Freeze-Drying	Creates porous scaffolds by freezing polymer solution and sublimating solvent.	Highly porous structure; good for drug/growth factor incorporation.	Requires careful parameter control; limited scalability.

**Table 5:** Summary of scaffold classes, manufacturing methods, and translational limitations in bone tissue engineering.

Scaffold Class	Key Materials/Manufacturing Methods	Translational Limitations
Ceramic-based	Hydroxyapatite (HA), β-TCP, bioactive glass, alumina, zirconia. Sintering, sol–gel, 3D printing	Brittle under cyclic/torsional loading; low tensile strength; poor machinability; limited degradation control; pure HA unsuitable for load-bearing without reinforcement [58,60,62,63].
Polymer-based (synthetic)	PLA, PLGA, PCL, PGA Electrospinning, 3D printing, freeze-drying, gas foaming	Acidic degradation byproducts may cause local cytotoxicity; poor osteoconductivity without surface modification; batch-to-batch variability in degradation profiles [64,65].
Polymer-based (natural)	Collagen, chitosan, gelatin, silk fibroin, hyaluronic acid. Freeze-drying, electrospinning, crosslinking	Weak mechanical properties unsuitable for load-bearing; rapid and unpredictable in vivo degradation; risk of pathogen transmission; limited scalability and reproducibility [64,65].
Polymer–ceramic composites	HA/PHBV, HA/PCL, β-TCP/PLA, bioactive glass/PLGA 3D-printing, electrospinning, compression molding, freeze-drying	Complex and poorly characterized degradation kinetics; potential byproduct cytotoxicity; inconsistent interfacial bonding between phases; limited long-term clinical datasets [58,59,63,64].
Metal-based	Titanium alloys (Ti-6Al-4V), stainless steel, cobalt-chromium, magnesium alloys, zinc alloys Selective laser sintering, 3D printing, casting	Stress shielding leads to periprosthetic bone resorption; corrosion and ion release toxicity in Mg/Zn alloys; non-degradable implants require revision surgery; fatigue failure risk [52,53,54,55,56,57].
Nano-hydroxyapatite (nHA) scaffolds	Nano-HA (<100 nm), nHA/polymer composites Co-precipitation, sol–gel, microwave synthesis, emulsion	Nanoparticle toxicity not fully characterized; agglomeration reduces uniformity; scale-up synthesis remains challenging; regulatory uncertainty for nanomaterials in clinical use [60,61].
Conductive polymer scaffolds	Polypyrrole (PPy), polyaniline (PANI), PEDOT, PEDOT: PSS Electropolymerization, chemical oxidation, electrospinning, 3D printing	Long-term in vivo stability and degradation profiles are poorly characterized; synthesis byproduct toxicity; limited large animal and clinical validation; complex regulatory classification [64].

## Data Availability

The original contributions presented in the study are included in the article, further inquiries can be directed to the corresponding author.
